# Supplementary material for: Association between Hematological Indicesand Disease Activity in Patients with Rheumatoid Arthritis Treated with Janus Kinase Inhibitors for 24 Weeks
Source: Medicina (Kaunas). 2022 Mar 15;58(3):426. doi: 10.3390/medicina58030426 (PMC8952825; doi:10.3390/medicina58030426)
Supplement: Supplementary file 1 [file medicina-58-00426-s001.zip › medicina-1612364-supplementary.pdf]

Table S1. Comparison of hematological indices between response group and non-response group at baseline.

	Response group (n = 104)	Non-response group (n = 11)	*p* value
ΔSII	921.9 (586.9, 1337.6)	873.2 (420.5, 2414.9)	0.909
ΔNHL score	0.282 (0.180, 0.379)	0.217 (0.136, 0.522)	0.992
ΔNLR	3.425 (2.222, 4.658)	2.576 (1.857, 6.979)	0.879
ΔPLR	170.8 (132.8, 234.6)	1.83.9 (112.6, 286.2)	0.917

P values were calculated by Mann-Whitney U test.

Abbreviation: SII, systemic immune-inflammation index; NHL, neutrophil-to-hemoglobin and lymphocyte ratio; NLR, neutrophil-to-lymphocyte ratio; PLR, platelet-to-lymphocyte ratio

Table S2. Comparison of changes in hematological parameters after treatment with JAK inhibitors for 24 weeks.

	Baricitinib (n = 64)	Tofacitinib (n = 51)	*p* value
ΔSII	-152.9 (-577.7, 152.3)	-315.8 (-755.2, 25.5)	0.220
ΔNHL score	-0.063 (-0.205, 0.025)	-0.093 (-0.186, -0.005)	0.636
ΔNLR	-0.809 (-2.390, 0.373)	-1.855 (-1.143, 0.104)	0.624
ΔPLR	0.934 (-53.779, 37.543)	-21.423 (-64.535, 23.189)	0.461
ΔHemoglobin (g/dL)	0.1 ± 1.1	0.3 ± 1.1	0.442
ΔPlatelet (x 10^3/μL)	24.7 ± 64.1	-13.7 ± 58.0	0.001
ΔNeutrophil (%)	-5.1 ± 10.5	-4.9 ± 12.3	0.940
ΔLymphocyte (%)	5.4 ± 9.6	5.3 ± 10.6	0.952
ΔNeutrophil (x 10^3/μL)	-0.9 ± 2.0	-1.3 ± 2.1	0.303
ΔLymphocytes (x 10^3/μL)	0.2 ± 0.5	0.1 ± 0.5	0.230

P values were calculated by Student *t*-test or Mann-Whitney U test.

Abbreviation: SII, systemic immune-inflammation index; NHL, neutrophil-to-hemoglobin and lymphocyte ratio; NLR, neutrophil-to-lymphocyte ratio; PLR, platelet-to-lymphocyte ratio
